# Genetic pass as a background of predictive, preventive, personalized medicine options and limitations

**DOI:** 10.1186/1878-5085-5-S1-A54

**Published:** 2014-02-11

**Authors:** Vladislav S Baranov

**Affiliations:** 1 Ott’s Institute of Obstetrics & Gynecology RAMS, St. Petersburg, Russia

## Objectives

Advances in human genome studies resulted in identification of numerous genes which, if mutated, provoke monogenic disorders of different kind or can contribute to a number of complex diseases (CD). While basic problems of monogenic disorders are already solved the genetic impact into CDs seems to be illusive so far in the most cases and remains a cumber-stone of currant PPPM. **Technology** Identification of candidate genes associated with complex diseases by **genetic testing, linkage studies and GWAS approach** resulted in relevant genetic panels specific for each of many CDs. However diagnostic value of these panels remains obscure for the most of known CDs so far. Individual genetic data contributing to human health as well as to the onset of a CD are quickly accumulated in Individual DNA -data bank, which could be interpreted as Individual Gene-Pass (IGP).

## Results

Associated gene studies already substantiated the issue of Gene-pass of Reproductive Health (GPRH). It consists of two mandatory parts of genetic testing results. The first - carriers testing in spouses for sever monogenic diseases. The second - the panels of candidate genes associated with preeclampsia, endometriosis, trombophilia, habitual abortions, diabetes, nerve tube defects chromosome non - disjunction, placenta insufficiency, as well as some pharmacogenetic tests elaborated for pregnant women. GPRH at use in our institute will be demonstrated. The genetic data together with relevant laboratory tests extrapolated to individual case history after sophisticated bioinformatic analysis (MDR test) are used for prospective individual risk evaluation.

## Outlook and expert recommendations

In spite on huge progress of whole genome sequencing it will never substitute the clinically important IGP incorporating only health meaningful genes and associations Suggested GPRH is the only one of many other IGPs already existing de facto. Genetic data supplemented with epigenetic information contributing to regulation of associated gene expression should be considered the next important step towards System Genetic Level of CD studies for PPPM purposes.

